# Supplementary material for: A source of entangled photons based on a cavity-enhanced and strain-tuned GaAs quantum dot
Source: eLight. 2024 Jul 24;4(1):13. doi: 10.1186/s43593-024-00072-8 (PMC11269457; doi:10.1186/s43593-024-00072-8)
Supplement: Supplementary file 1 — Supplementary material 1. [file 43593_2024_72_MOESM1_ESM.docx]

Supplementary Information to

A source of entangled photons based on a cavity-enhanced and strain-tuned GaAs quantum dot

**S1 | Device Fabrication**

**Simulations of the cavity**

A finite difference time domain (FDTD) based commercial software solution is used to simulate the performance of circular Bragg resonators (CBRs). The performance of Al_2_O_3_ as a material system for the oxide in the broadband back reflector was studied, with the results in extraction efficiency and Purcell factor provided in Fig. S1.


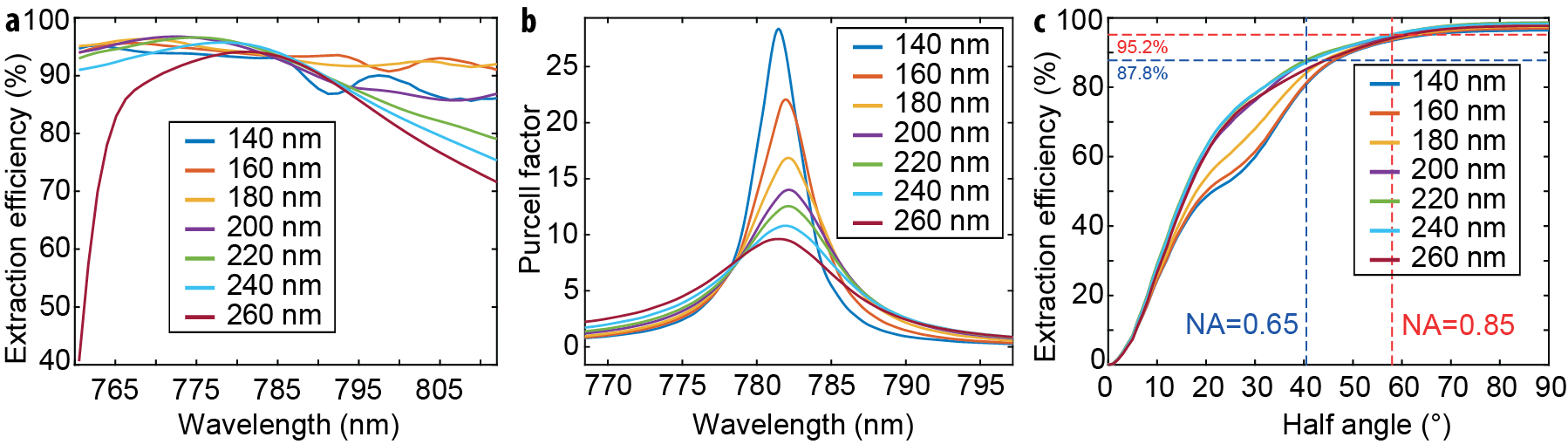


**Figure S1 |** FDTD simulated data for various Al_2_O_3_ layer thicknesses (indicated in (a) and (c)) showing **(a)** the extraction efficiency and **(b)** the Purcell factor as a function of wavelength. **(c)** A Gaussian intensity distribution is indicated by a slowly varying extraction efficiency at high collection angles.

**Deposition of the back reflector**

We employ an atomic layer deposited (ALD) thin film of amorphous Al_2_O_3_ with a refractive index n = 1.64 at a wavelength of 780 nm that has a nominal thickness of 160 nm. This value is chosen to optimize the performance of the structure, as evaluated via finite-difference time-domain numerical simulations (see Section S1). The layer is grown on a sample with GaAs QDs on a GaAs substrate at a temperature of 200 °C in 1600 cycles, which consist of alternate pulses of trimethylaluminum (TMA) and water with pulse lengths of 15 ms each, separated 8 s in time. From an atomic force microscope (AFM) image we can extract a root mean square (RMS) surface roughness of 0.5 nm of this layer, as shown in Fig. S2 (a). The actual layer thickness was found to be 197 nm.

On top of the oxide layer, a 150 nm thick film of gold is thermally evaporated in a physical vapor deposition (PVD) process. This layer serves as a broadband reflector. To ensure sufficient adhesion a 3 nm layer of chromium is deposited prior to the Au by e-beam evaporation. Deposition rates are tracked in-situ using a quartz crystal resonator and regulated using a PID controller to achieve 5 Å/s for Au and 2 Å/s for Cr. An AFM surface scan yields an RMS roughness of 1.8 nm.


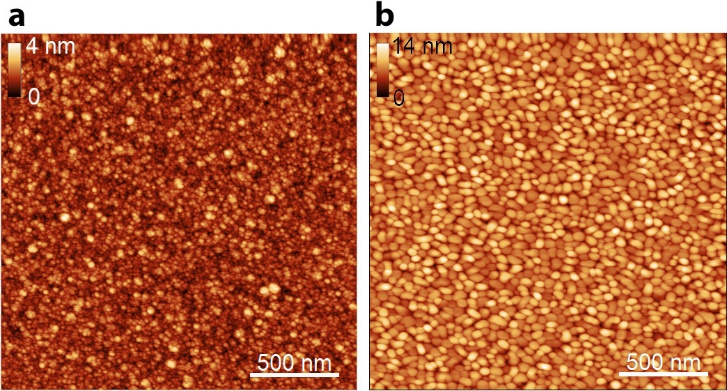


**Figure S2 | (a)** AFM scan of the Al_2_O_3_ layer deposited with ALD. **(b)** AFM scan of the Au layer deposited with PVD.

**Flip-Chip stack bonding**

Nanomembranes on a micro-machined piezo substrate can suffer from cracking when cooled to cryogenic temperatures when directly bonded to the substrate which makes an intermediate carrier sheet necessary for additional structural stability. We choose GaAs as “carrier sheet” to have the same thermal expansion coefficient of the QD membrane material and avoid mechanical stress to the membrane during the curing process (see below). In the first step, SU-8 is applied onto the gold surface of the sample with a brush and cured on a hot plate at 65 °C for 2 min and 95 °C for 5 min to evaporate solvents. Next, the temperature is raised to 130 °C to mobilize the solvents in the resist and contact is made with the polished surface from an equally sized piece of a bare GaAs wafer with a thickness of 350 μm utilizing a die bonding machine. Elevating the temperature to 230 °C for 10 min sparks crosslinking of the polymers in the resist, strongly bonding the two chips together.

Subsequently, the two-piece stack is lapped down from the GaAs new substrate side until this reaches a thickness of about 50 µm. For this step, the sample substrate acts as handle wafer. The stack is lapped parallel to the surface by a mixture of powdered Al_2_O_3_ and water as an abrasive.

By repeating the same bonding sequence, the carrier sheet is bonded onto the six-legged piezo such that it is stacked in between the upside-down sample and the piezo substrate.

**Multi-step wet chemical etching**

To remove the substrate used to grow the QDs, a four-step wet chemical back-etching process is employed, including a phosphorous acid solution for fast etching, timely changed to citric acid solution for selective etching stopping at the sacrificial layer and a subsequent removal of the sacrificial layer with hydrofluoric acid. Depending on environmental circumstances that cannot be controlled, residuals from the HF etching may possibly appear. These can be removed in potassium hydroxide.

For protecting the carrier sheet and the piezo substrate, the sample is glued to a Si chip carrier and protected on the sides using photoresist which is cured at 95 °C for 10 min on a hot plate.

The solutions are always freshly prepared. Volumetric mixing ratios include a 3:7 ratio of 85 % H_3_PO_4_ and 30 % H_2_O_2_ yielding a fast etch rate of approximately 5 µm/min when balancing the mixture for 15 min prior to etching and agitating the solution with a magnetic stirrer spinning at 150 rpm. The etching of the residual GaAs is obtained with a 1:4 ratio of H_2_O_2_ and powdered C_6_H_8_O_7_ dissolved in a 1:1 mass ratio in water for slow but selective etching with a rate of 0.5 µm/min. The latter etchant is extremely selective against GaAs^1^ and stops at the Al_0.75_Ga_0.25_As layer. To remove the 400 nm thick sacrificial layer, etching in 10 % concentrated HF lasts only a few seconds. Residue from the sacrificial layer appearing on the sample surface are removed by dipping the sample in 44% KOH for 200 s.

**Electron beam lithography and deposition of markers**

To find the positions of QDs, a frame of reference is introduced, realized as metallic markers that are patterned using electron beam lithography (EBL). A film of 400 nm thick AR-P 6200.13 is spin-coated onto the sample surface and cured at 150 °C for 1 min on a hot plate. To avoid charging during the patterning, a 42 nm thick protective coating of AR-P 5090.02 is spun on the previous resist and cured at 90 °C for 1 min.

The design of the reference markers is patterned in EBL with an acceleration voltage of 30 kV, an aperture of 20 µm, and a dose of 65 µC/cm². The machine used is a Raith eLine system. After patterning, the protective resist is removed in a 1 min bath in de-ionized water and the e-beam resist is developed in AR 600-546 for 1 min. Development is stopped in isopropanol.

A stack of 50 nm Cr followed by 50 nm Au and again 50 nm Cr is deposited on the sample with the developed resist using PVD as described above. The use of a high atomic number element, such as Au, enables the detection of the markers using EBL with 100 kV acceleration voltage for deterministic patterning of photonic structures, while the symmetrized Cr layers serve for preventing the markers to curl, as well as for providing optical contrast with respect to the broadband reflector of the nanomembrane when illuminating with infrared (IR) light in the cryogenic microscopy setup used to measure single QD positions, see below. The reference markers are finished after a lift-off process in an alternate bath of anisole and acetone, as well as sonication.


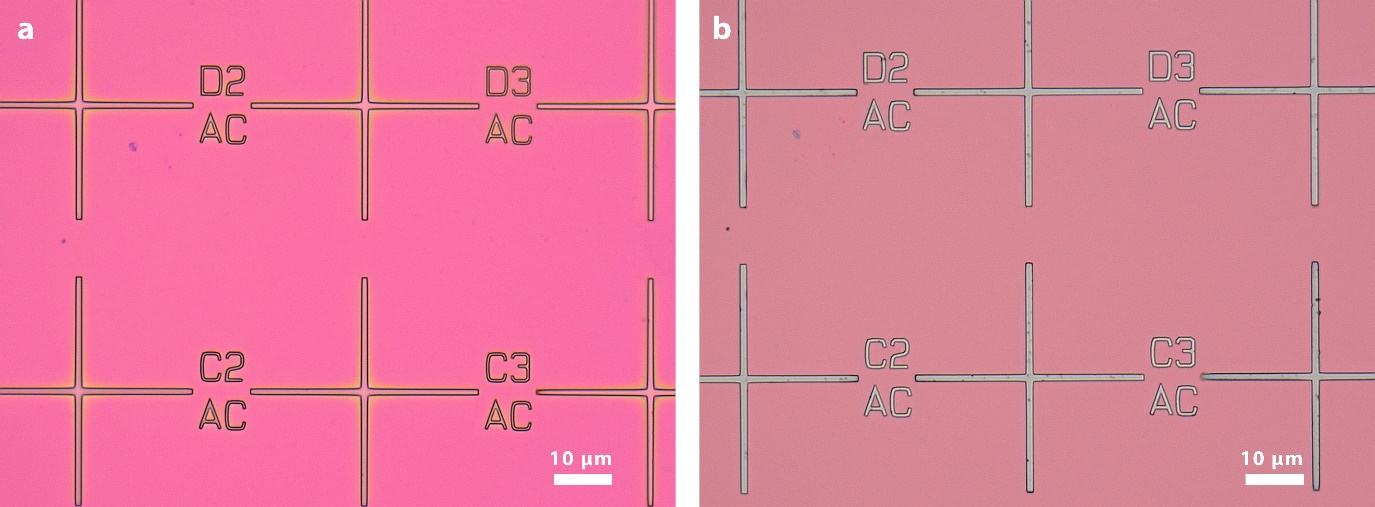


**Figure S3 | Reference markers with a periodicity of 50 µm** **(a)** after developing of the e-beam resist and **(b)** after metal evaporation and lift-off.

**Imaging and QD position mapping**

To acquire the QD positions through their photoluminescence (PL), the sample is placed in a continuous-flow cryostat and cooled with liquid He and imaged with a custom-built microscope, see sketch in Fig. S4a. To avoid contamination from the cryostat on the sample surface hindering subsequent processing steps, a protective layer of polymethylmethacrylate (PMMA) is applied. An 810 nm infrared (IR) light emitting diode (LED) illuminates the surface while a 470 nm blue LED simultaneously excites PL from the QDs. Metallic reference markers have Cr on the top, absorbing incident IR light and providing contrast to the reflective background (Au mirror placed below the AlGaAs membrane, which is transparent to the used IR light). QD PL emission is collected with a dichroic mirror with a cut-off wavelength of 650 nm. A glass-corrected near-IR optimized objective with a numerical aperture of 0.85 is used together with an achromatic lens doublet with a focal length f = 100 mm to project an image with a magnification of 57 onto a monochromatic CMOS active pixel type solid-state image sensor with a pixel size of 2.9 x 2.9 µm² and a resolution of 1936 x 1096 pixels, operated with low gain and an exposure time of 1 s. Spectroscopy on single QDs is performed by using a 532 nm green diode laser and diverting the signal toward a spectrometer. Using the PL emission of QDs for characterizing the point spread function (PSF) of the imaging system under the same conditions as used to record images, we find that light from the QDs appears as Airy discs with a full width at half maximum (FWHM) of 520(25) nm (statistical value of 372 QDs), which corresponds to a value 1.11(5) larger than the anticipated value of the diffraction limit of the objective lens:

$\mathrm{FWHM}_{\mathrm{lateral}}=0.51 \frac{\lambda}{\mathrm{NA}}$ .


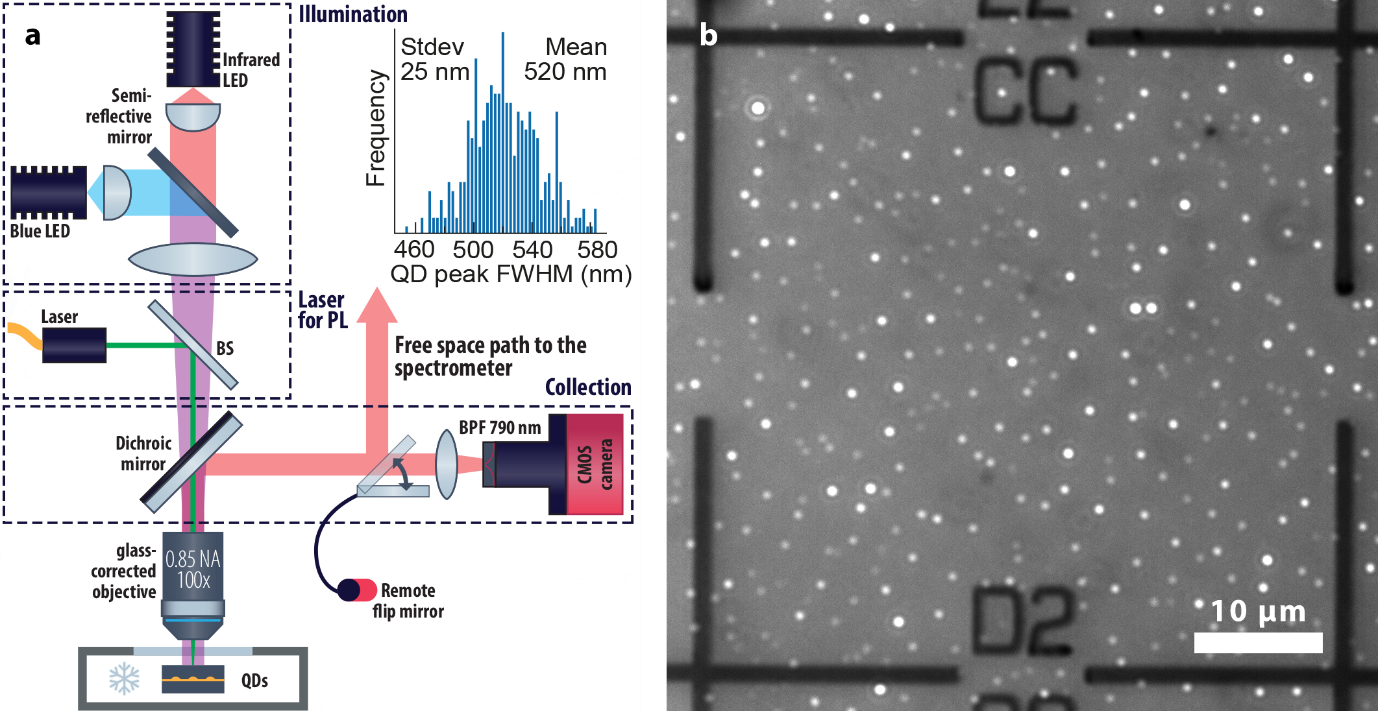


**Figure S4 | (a)** A sketch of the PL imaging setup with IR and blue LEDs for surface illumination and PL excitation, a fibre-coupled laser for µ-PL of single QDs and collection using a dichroic mirror and a band-pass filter for imaging on a monochromatic CMOS camera. The inset shows a histogram of the FWHM of the central peak of Airy discs corresponding to the PL of different QDs. The mean value of the FWHM is 520(25) nm. **(b)** Example of image of excited QDs and illuminated sample surface.

QD positions with respect to the frame of reference of the metallic markers are extracted from the image by using a script for numerical image processing including a Hough transformation for finding the markers, a Gaussian fit perpendicular to the lines of the marker to refine their detection, as well as a peak finder with 2D Gaussian fits for finding the peak position of each single QD emission spot.

A statistical method is employed to estimate the random and numerical error of QD positions with respect to the reference markers. To generate data, 30 images of the same marker field are taken, and the position mapping script is applied to each of them, yielding the arithmetic mean position as well as the standard deviation for x and y coordinates for each QD position. The standard deviation of the Euclidian distance to the mean can be calculated through the addition of the variances in both directions, as

$\sigma_{r}= \sqrt{\sigma_{x}^{2}+\sigma_{y}^{2}}$ ,

which provides one $\sigma_{r}$ for each QD position. Those deviations are counted in a histogram binned into 1 nm columns. A fit of such data containing 284 data points using a log-normal distribution shows a peak at 18 nm, whereas the median of the data set is 22 nm. With numerical image pre-processing such as thresholding, brightness control and rescaling intensity values, the peak of the log-normal fit on 280 data points can be reduced to occur at 10 nm, while the median is 15 nm.


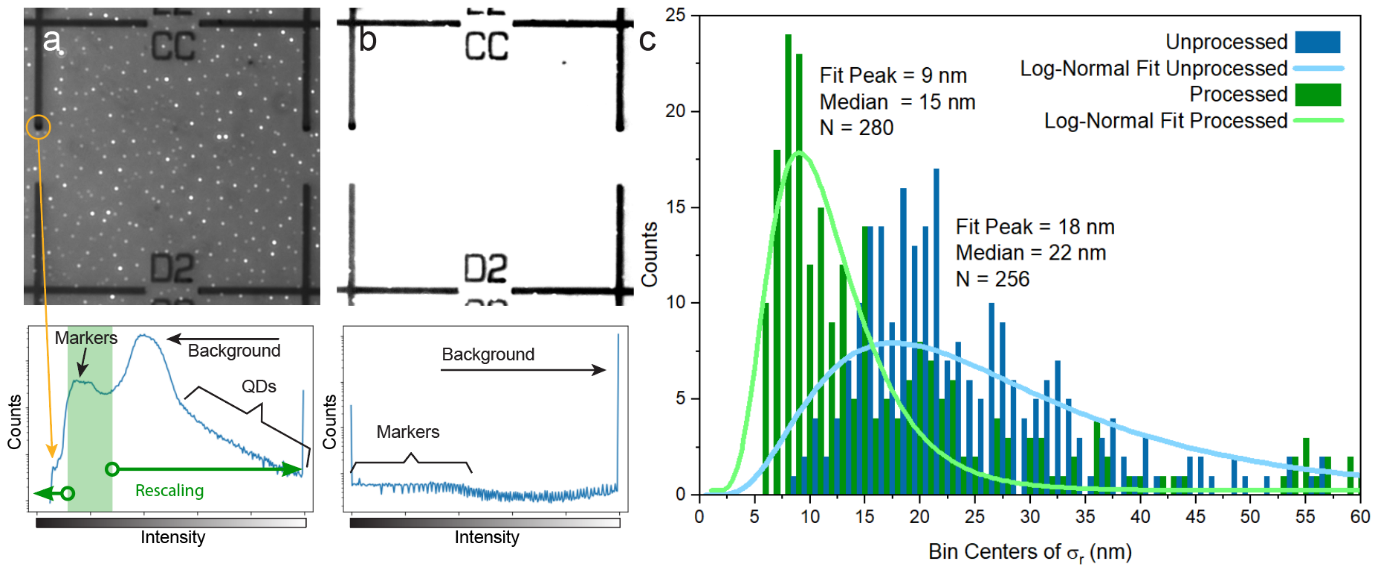


**Figure S5 | (a)** Recorded image of markers and QDs with its corresponding intensity histogram in logarithmic scale (bottom). Dark/bright pixels in the image correspond to bins in the left/right part of the histogram, as indicated by the intensity scale. Pixels corresponding to the markers yield the peak marked in green in the histogram with a side peak to the left stemming from dark spots in the image of markers. **(b)** Through rescaling the green area of (a) to full range, QDs which are parasitic in the marker detection process can be removed, and the contrast of markers and background can be maximized, as well as the markers’ internal structure reduced. **(c)** Histogram of the standard deviations of the Euclidian distance $\sigma_{r}$ to the mean of *N* QD positions. “Unprocessed” corresponds to marker detection performed in the image from panel (a), whereas “Processed” belongs to marker detection performed in the image of panel (b).

**Calibration of cavity mode position**

Due to layer-thickness gradients occurring during the epitaxial growth and oxide deposition, the cavity mode of a given CBR design shifts in its spectral position depending on the lateral position of the CBR on a sample. Therefore, a calibration of the central disc radius of the CBR turned out to be necessary. Light from a white light source is focused to a spot impinging in the centre of a CBR structure at room temperature. The reflected signal is normalized by the reflection of the spot outside of such a structure on the nanomembrane. Reflectivity spectra are shown in Fig. S6 and the dependency on the centre disc radius is extracted. To match the mode position with the emission of QDs at cryogenic temperatures, a blue-shift of the cavity mode of 10 nm needs to be considered, as extracted by temperature-dependent measurement of the cavity mode, not shown here.


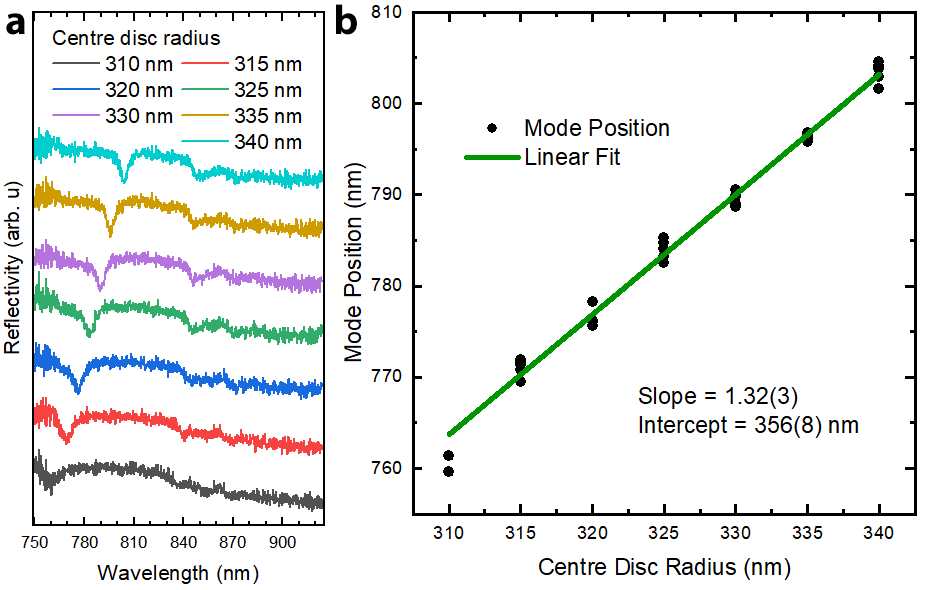


**Figure S6 | (a)** µ-reflectivity spectra of several structures fabricated for calibration on the same chip. The mode is visible as a reflectivity dip moving towards higher wavelengths for larger centre disc radii. **(b)** A linear fit is used to recreate the desired wavelength.

**Deterministic fabrication of CBR structures**

CBR cavities are fabricated deterministically on QD sites with tailored mode positions for certain single QDs. If no spectral information of a QD is available, CBR cavities are equally distributed with target mode positions at X + 1 nm, X + 3 nm, and X - 1 nm, where X is the mean spectral position of the excitonic transition on the sample. Position and geometry of CBR structures are provided to a Jeol JBX-9300FS 100 kV EBL system which aligns to the frame of reference induced on the sample through the deposited metallic markers. The intermediate Au layer interacts with the electron beam providing contrast to the semiconductor surface. The EBL system aligns the internal coordinate system using perpendicular line scans on the lines of the marker crosses. The design is then transferred onto an approximately 550 nm thick layer of PMMA resist serving as a mask for reactive ion etching (RIE) using an inductively coupled plasma (ICP) source. CBR structures are etched using a Cl_2_ and Ar mixture (1.7 sccm to 18 sccm) with a 10 mTorr pressure, a radio-frequency (RF) power of the substrate electrode of 50 W, an ICP power of 150 W, and a bias voltage of 250 V yielding a roughly estimated etch rate of 85-100 nm/min.


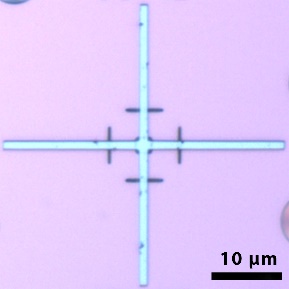


**Figure S7 |** Four-line scans of the e-beam perpendicular to the marker cross lines and a square scan in the centre of the cross. Exposed areas of the resist due to the scan are developed and the underlying AlGaAs layer dry-etched as well.

To estimate the precision of EBL-assisted deterministic patterning we compare the position of detected QDs with the position of deterministically patterned Au discs. On an as-grown sample, i.e., no nanomembrane, approximately 100 QD positions are recorded per each of 221 marker fields to pattern gold discs with radii distributed among 150 nm and 500 nm on QD sites using the Raith eLine 30 kV EBL system. A subsequent metallization, lift-off, and imaging of all Au discs through reflectivity yields the positions of those discs with respect to the reference markers using the same script as used for QDs. An image of a marker field with 100 detected QDs, an image of the same marker field but with Au discs, as well as a histogram of the count of Euclidian distances Δ*r* between centres of corresponding Au discs and QDs is provided in Fig. S8, yielding an estimated patterning accuracy of 33 nm (position of the peak of the Log-Normal fit), while the median of Δ*r* values is 43 nm.

**
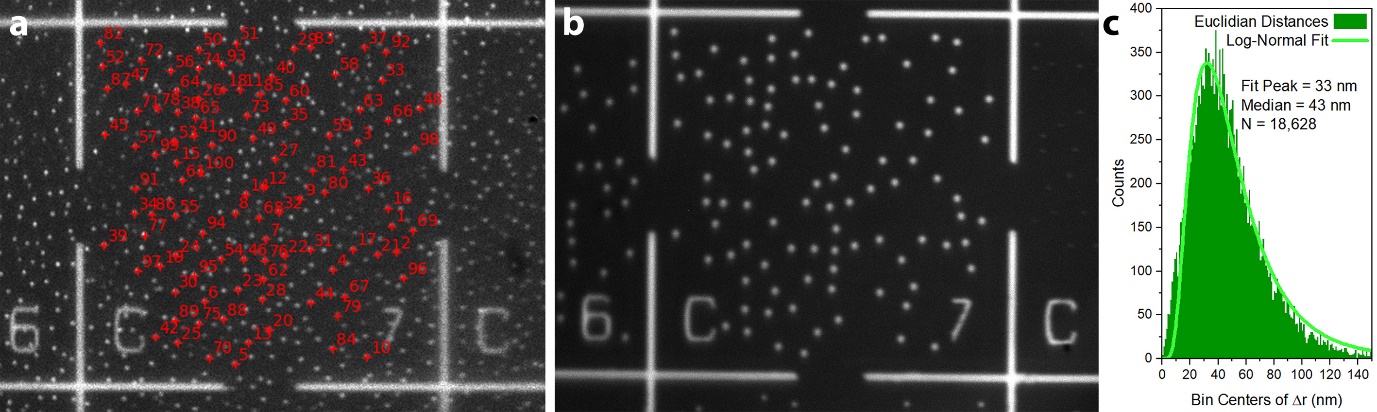
**

**Figure S8 | (a)** Au markers reflecting the IR LED and PL of QDs excited by a blue LED. A set of 100 QDs was recorded for each marker field. **(b)** Au markers and Au discs on pre-recorded QD sites, both reflecting the IR LED. **(c)** Histogram of the Euclidian distances Δ*r* between the centers of QDs and Au discs using 18,628 data points.

**S2 | Cavity mode characterization at low temperature**

The mode of a cavity is characterized in a cryostat at low temperature (5K) by focusing a white halogen lamp (3000K) on the sample through a 0.5 NA aspheric lens and collecting light from the centre of the cavity with a single mode fibre. The collected spectra are normalized with the flatfield response of the system obtained by shining the same light on a highly reflective part of the sample (namely the unprocessed membrane). The two reflectivity spectra for the cavities containing QD2 and QD4 discussed in the main text are shown in Fig. S8, panels a and b, respectively. The emission from the QD states excited by the white lamp is also visible in the reflectivity spectra. The detuning from the cavity mode centre influences the magnitude of the Purcell factor. As expected, a lower detuning, as for QD2 in panel a of Fig. S9, produces a higher Purcell factor.

From a Fano fit of the cavity mode it is possible to extract the quality factor (Q) of the cavity. The extracted values, 115(1) and 104(1) for the CBRs with QD2 and QD4, respectively, are slightly lower than the expected values for this type of structure (150) but comparable in order of magnitude.

**
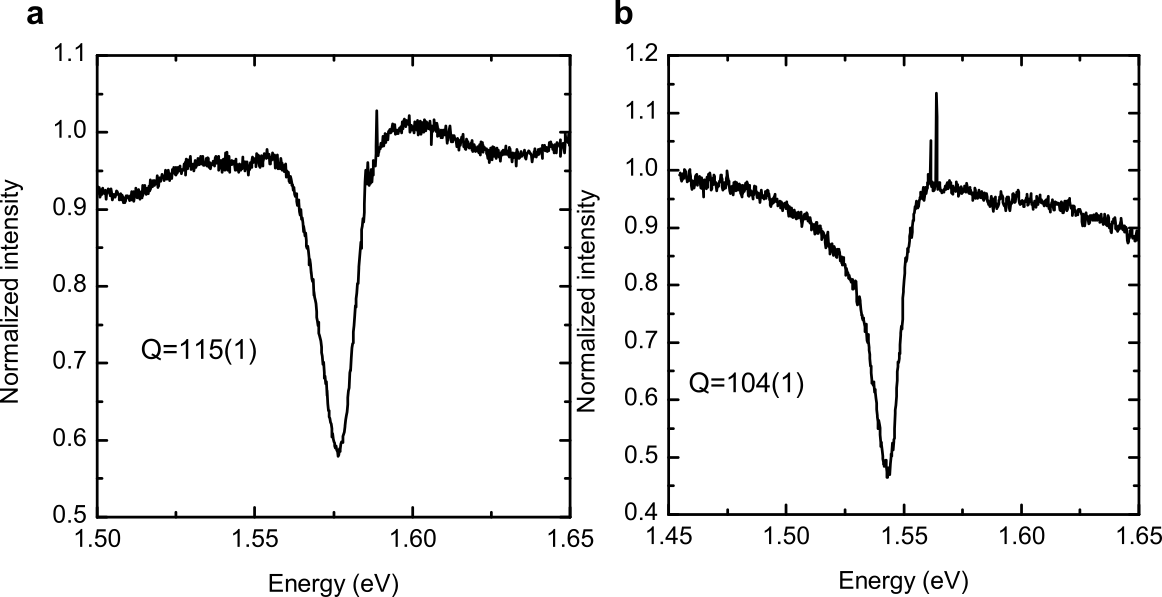
**

**Figure S9 |** Normalized low temperature reflectivity spectra of the CBRs containing QD2 (a) and QD4 (b). The quality factor is calculated from a Fano resonance fit of the cavity mode dip^2^.

**S3 | Lifetimes**

The lifetime measurement setup and conditions are described in detail in the Methods section of the main text. In Fig. S10 we report the time-resolved PL traces for QD2 and QD4, panel (a) and (b) respectively. The values of the lifetime for X and XX are extracted by fitting the experimental curves with an exponential decay and instrument response function (IRF) convolution and are reported in figure.


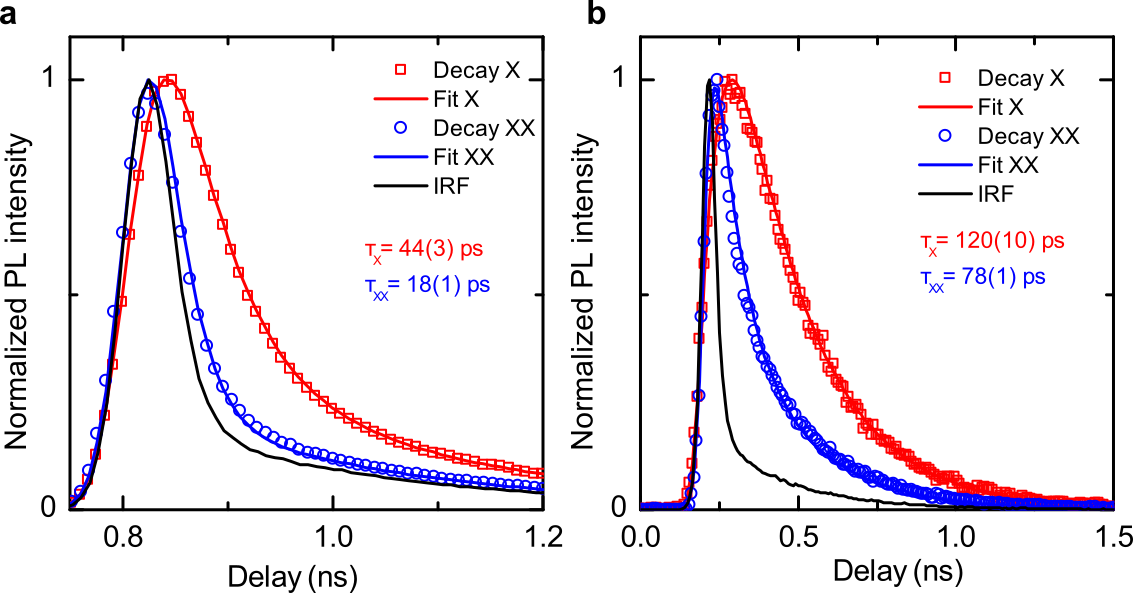


**Figure S10 |** Time-resolved photoluminescence of the X (red squares) and XX (blue circles) transition intensities from QD2 **(a)** and QD4 **(b)**, and instrument response function (IRF) (black solid line) of the setup. The lifetime values are obtained with a fit (solid lines) convoluting the IRF with the exponential decay functions expected from the radiative cascade.

**S4 | Rabi oscillations**

The signal of the X line of the brightest QD in the sample, excited with TPE, is coupled into a multimode fibre and sent to a silicon avalanche photodiode (APD). The number of counts per second on the APD is recorded while increasing the power of the laser used for TPE. The Rabi oscillations in the intensity are a clear signature of the resonant excitation.


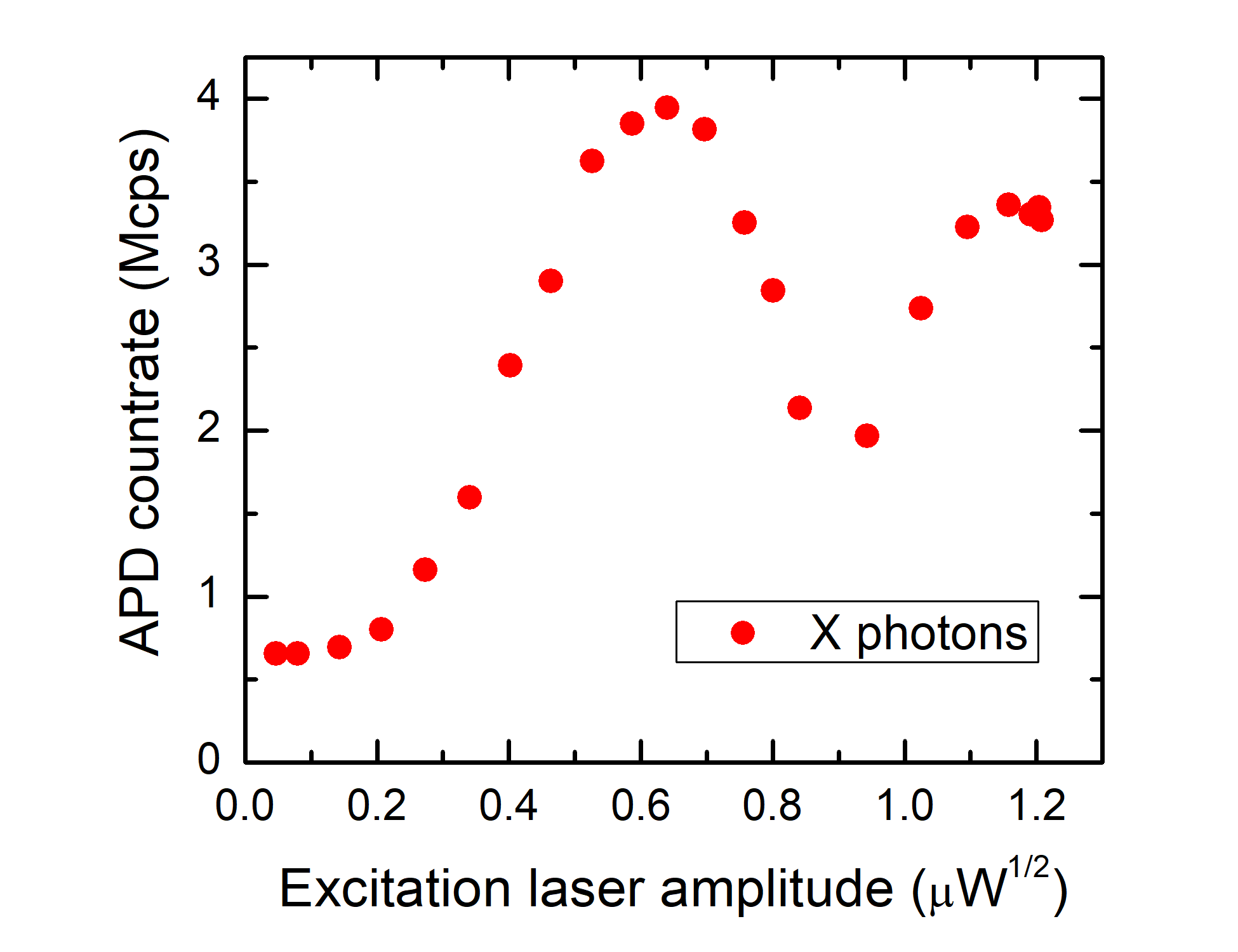


**Figure S11 |** Count rates on the avalanche photodiode for the X transition coupled in a multimode fibre as a function of the excitation power of the two-photon resonant laser. The non-zero intensity at null laser power is attributed to above-band excitation due to the white light used to neutralize the QD.

**S5 | Extraction efficiency**

To calculate the extraction efficiency, we measure the photon rates at the APDs at π-pulse and quantify all the other figures that contribute to the observed photon rates: the efficiency of the excitation, the transmission through the optical elements of the setup, the fibre coupling efficiency, and the efficiency of the detectors.

The measured photon rates of the X and XX are corrected to isolate only the contribution from the two-photon cascade: we subtract from the counts of the X channel the number of photons which are emitted by above band excitation from the white halogen lamp shining on the sample to neutralize the QD and enable the TPE (see the non-zero counts at null laser power in Fig. S11), while we subtract from the XX channel counts the contribution from an emission line close to the XX, whose ratio is calculated from the spectrum of the signal going to the APD.

The pair emission probability $\eta_{pair}$ is the combination of the preparation fidelity of the TPE $\eta_{prep}$, i.e., the probability that the laser pulse excites the QD when it is sitting in its charge-neutral ground state, and the blinking ratio $\eta_{blink}$, i.e., the fraction of time that the QD is optically active (in its charge-neutral ground state). $\eta_{pair}$ is calculated from the corrected, see previous paragraph, single photon count rates of the X and XX channels ($\tilde{R}_{X}$ and $\tilde{R}_{XX}$) and the coincidences between the two channels ($n_{c}$) multiplied by the laser repetition rate (the rates were all measured in single mode fibres):

$$\eta_{pair}= \frac{\tilde{R}_{X}\cdot\tilde{R}_{XX}}{n_{c}\cdot R_{laser}}=\frac{1.75\left( 1 \right) Mcps\cdot1.98\left( 1 \right) Mcps}{0.155\left( 1 \right) Mcps\cdot80 Mcps}=0.279\left( 3 \right)$$

and contains also any non-radiative channel and the quantum efficiency of the process. By estimating the average blinking fraction $\eta_{blink}=0.29$ from long time range $g^{(2)}$ measurement we can calculate the preparation fidelity as $\eta_{prep}=0.96$.

The losses on the bulk optics were estimated via direct measurement of the power of a laser beam with the same wavelength of the QD emission passing along the same path as the signal from the QD. The components comprise an aspheric lens, two windows of the cryostat, a two-lenses telescope to reduce the beam size, a 90:10 beam splitter, two notch filters, a long pass 750 nm filter, the two volume Bragg gratings (VBG) notch filters used to reflect only the X and XX lines, four metallic high-reflection mirrors and one dielectric mirror. The setup transmission was measured as $\eta_{setup}^{X}=0.60(2)$ and $\eta_{setup}^{XX}=0.62(2)$.

The multimode fibre coupling efficiency, $\eta_{FC,MM}=0.929(3)$, was estimated by measuring the laser power before the fibre coupler and at the exit of the fibre. The single mode fibre coupling efficiency was derived by comparing the count rates of the detectors in multimode fibre and in single mode fibre measured under the same conditions.

To calculate the actual efficiency of the detectors $\tilde{\eta}_{det}$ we must correct the efficiency given by the manufacturer, $\eta_{det}=0.46$, for the non-linearity due to the detector dead time $\tau_{det}=34 ns$. The correction factor is obtained by observing the change in intensity of the signal while placing neutral density filters with different optical density in front of the detectors, calibrated at the correct wavelength. The corrected efficiency values obtained are $\tilde{\eta}_{det}^{X}=0.379(2)$ and $\tilde{\eta}_{det}^{XX}=0.410(2)$. If we estimate the expected detector efficiency based on the observed count rate and on the dead time, including a first order non-linearity correction:

$$\tilde{\eta}_{det}=\eta_{det}\cdot\left( 1-\tau_{det}\cdot R_{QD} \right)$$

we observe a difference with respect to our experimental estimate. This discrepancy is arguably to be ascribed to the blinking of the QD. We model the blinking using a telegraphic model in which the QD either emits light (on) or does not emit light (off), staying on for an average time $\tau_{blink}$ and for a fraction of total time $\eta_{blink}$. Due to this effect, if $\tau_{blink}$ is higher or comparable to $\tau_{det}$, the detectors receive a photon flux which is much higher than the average value measured, when the QD is on, and no photons when the QD is off. The non-linear response of the detector must then be corrected for the actual value impinging on the sensor during the on-time of the QD, also considering the interplay between the deadtime of the detector $\tau_{det}$ and the switching time of the QD $\tau_{blink}$. The detector efficiency can then be corrected in the case of a blinking QD by adding a multiplicative factor on the actual count rate:

$$\tilde{\eta}_{det}=\eta_{det}\cdot\left( 1-\tau_{det}\cdot\frac{R_{QD}}{c_{blink}} \right)$$

Here, $c_{blink}$ has an average value of $\sim0.7$, which is higher than the estimated $\eta_{blink}$. This is because $\tau_{blink}=\sim60 ns$ is close to $\tau_{det}$ so that only the slower blinking components contribute to the increase in the effective photon flux seen by the detectors. This figure can be associated with the drop in coincidences at the delays longer than the detector dead time in the intensity autocorrelation function. By correcting the measured rate at the APDs with $\tilde{\eta}_{det}$ we can also obtain the actual average photon flux impinging on the APDs in multimode fibre as $R_{QD,MM}=\frac{R_{APD,MM}}{\tilde{\eta}_{det}}=9.6(1)$ Mcps.

The extraction efficiency can be then finally calculated from the formula:

$$R_{APD}=R_{laser}\cdot\eta_{pair}\cdot\eta_{ext}\cdot\eta_{setup}\cdot\eta_{FC}\cdot\tilde{\eta}_{det}$$

as:

$$\eta_{ext}=\frac{R_{APD}}{R_{laser}\cdot\eta_{pair}\cdot\eta_{setup}\cdot\eta_{FC}\cdot\tilde{\eta}_{det}}$$

**Table S1 |** Measured photon rates and efficiencies of the different parts of the experimental setup for the X and XX emission channels.

|  | $\tilde{R}_{APD,SM}$ (Mcps) | $\tilde{R}_{APD,MM}$(Mcps) | $R_{laser}$ (Mcps) | $\eta_{pair}$ | $\eta_{setup}$ | $\eta_{FC,SM}$ | $\eta_{FC,MM}$ | $\tilde{\eta}_{det,SM}$ | $\eta_{ext}$ |
| --- | --- | --- | --- | --- | --- | --- | --- | --- | --- |
| X | 1.75(1) | 3.13(1) | 80 | 0.279(3) | 0.60(2) | 0.511(4) | 0.929(3) | 0.379(2) | 0.67(3) |
| XX | 1.98(1) | 3.52(1) |  |  | 0.62(2) | 0.508(5) |  | 0.410(2) | 0.69(4) |

**S6 | FSS erasure**

**
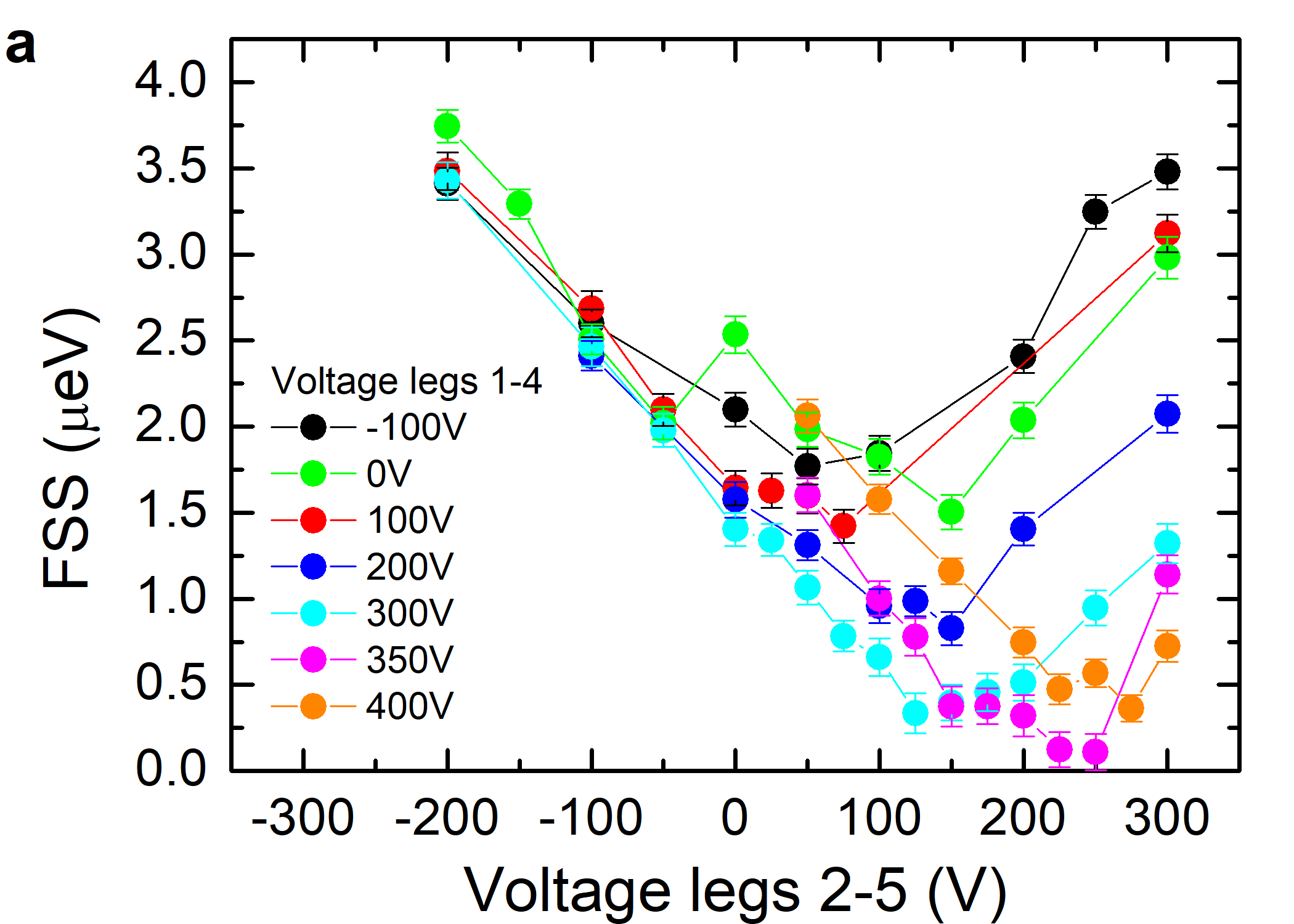

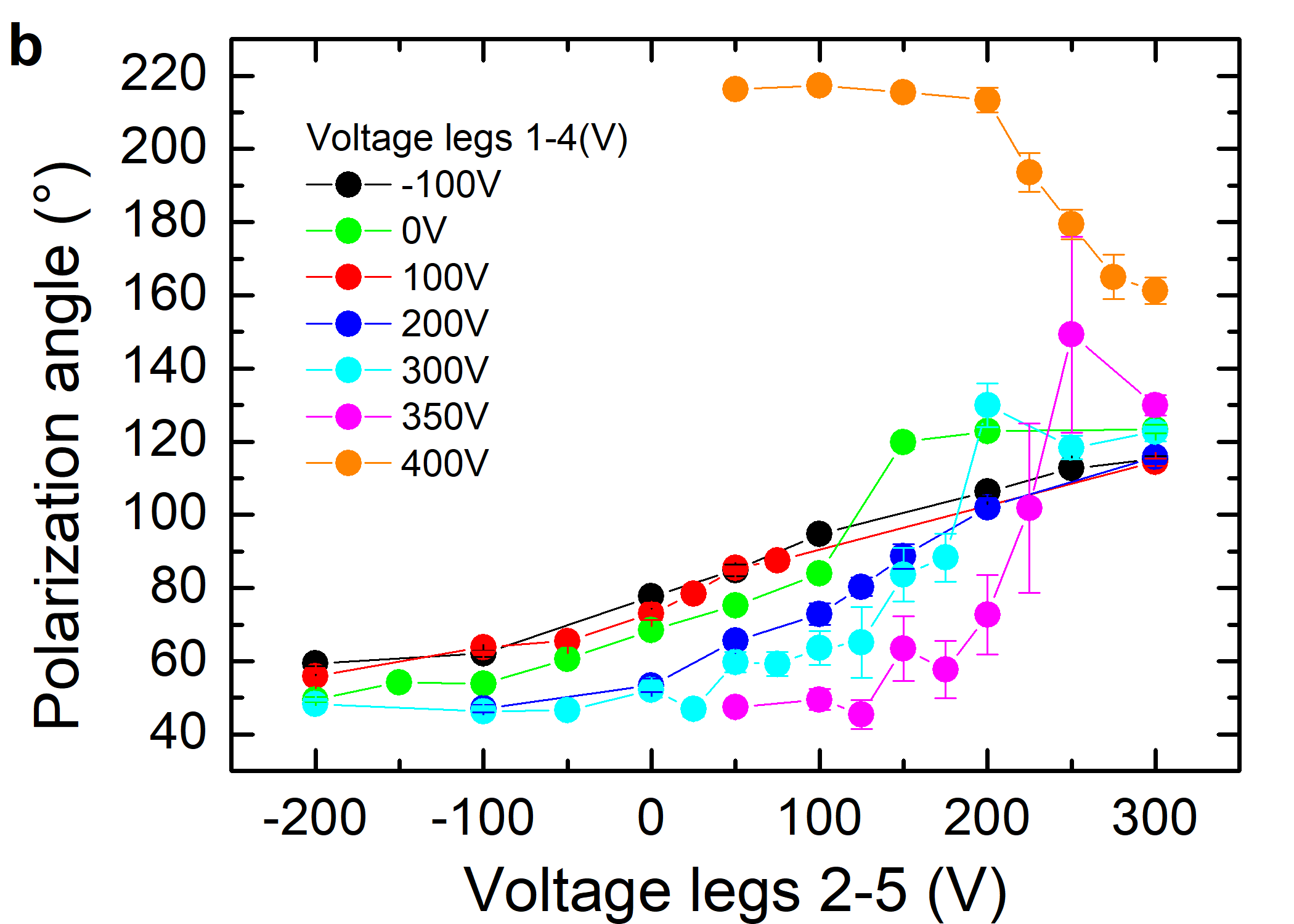
**

**Figure S12 | (a)** Fine structure splitting (FSS) and **(b)** polarization angle vs applied voltage on the piezoelectric actuator legs of QD2.

The FSS erasure procedure was replicated on QD2 by exchanging the sweep order of legs 1-4 and 2-5. Fig. S12 shows the amplitude of the FSS and the polarization angle for several sweeps on legs 2-5 while changing the voltage of legs 1-4. The voltage sweep corresponding to the minimum of the FSS is the pink one. The minimum value is obtained for a voltage on legs 1-4 of 350V and 250 V on legs 2-5, which are compatible with the conditions found previously for a minimum on the same QD, as shown in Fig. 3a of the main text. Increasing the voltage of legs 1-4 beyond this point produces a flip in the angle of the polarization (orange curve), confirming that the minimum condition is surpassed^3^.

**References**

1. Tong, M. *et al.* A comparative study of wet and dry selective etching processes for GaAs/AIGaAs/lnGaAs pseudomorphic MODFETs. *J. Electron. Mater.* **21**, 9–15 (1992).

2. Krieger, T. M. *et al.* Postfabrication Tuning of Circular Bragg Resonators for Enhanced Emitter-Cavity Coupling. *ACS Photonics* **11**, 596–603 (2024).

3. Trotta, R. *et al.* Universal Recovery of the Energy-Level Degeneracy of Bright Excitons in InGaAs Quantum Dots without a Structure Symmetry. *Phys. Rev. Lett.* **109**, 147401 (2012).
